# Variant selection in surface martensite

**DOI:** 10.1107/S160057671701398X

**Published:** 2017-10-20

**Authors:** Annick P. Baur, Cyril Cayron, Roland E. Logé

**Affiliations:** a Laboratory of Thermomechanical Metallurgy–PX Group Chair, Ecole Polytechnique Fédérale de Lausanne, Switzerland

**Keywords:** surface martensite, variant selection, phenomenological theory of martensite crystallography (PTMC), continuous f.c.c.–b.c.c. distortion

## Abstract

A significant variant selection is reported in isothermal martensite formed on the surface of an Fe–30% Ni sample. The selection phenomenon is modelled using different descriptions of the martensitic phase transformation. In particular, matrices based on the phenomenological theory of martensite crystallography, the Jaswon and Wheeler distortion, and the continuous face centred cubic–body centred cubic distortion are compared. All descriptions allow good predictions of the variant selection. However, the Jaswon and Wheeler distortion and the continuous distortion better account for other features of the surface martensite, such as the {225}_γ_ habit plane and the accommodation mechanism by twin-related variant pairing.

## Abstract 2

Variant selection is reported in martensite formed on the surface of an Fe–30% Ni sample. Predictive models of the phenomenon based on different crystallographic descriptions of the transformation are proposed and compared.

## 1. Introduction   

Variant selection in martensitic steels is a well documented phenomenon because of its implications for the industrial processing of steel. It is reported when stress is applied to the material either before (Wittridge & Jonas, 2000 ▸; Miyamoto *et al.*, 2012 ▸) or during transformation (Gey *et al.*, 2005 ▸; Mishiro *et al.*, 2013 ▸). In this study, we show a significant variant selection in isothermal surface martensite formed on the free surface of an as-cast Fe–30% Ni sample without any applied stress. The formation of surface martensite above the bulk martensitic start temperature has been observed since the 1950s in different types of steels, for example in high-carbon steels, iron–nickel alloys and stainless steels (Klostermann & Burgers, 1964 ▸; Klostermann, 1972 ▸). More recently, spontaneous martensitic transformation around a free surface created by focused ion beam milling in retained austenite grains of stainless duplex steel was also noticed (He *et al.*, 2014 ▸). However, to our knowledge, variant selection in surface martensite has never been reported. The present study shows this phenomenon and proposes a predictive model, based on the maximization of the extension of a material fibre oriented along the normal to the sample’s free surface. This model is used to compare different crystallographic descriptions of the transformation. In particular, we consider the invariant plane strain and the invariant line strain based on the phenomenological theory of martensite crystallography (PTMC) (Bowles & Mackenzie, 1954 ▸), the Jaswon and Wheeler distortion (Jaswon & Wheeler, 1948 ▸), and the continuous distortion associated with the Kurdjumov–Sachs orientation relationship (Cayron, 2015 ▸). The study shows that all four descriptions of the transformation are able to predict correctly the trend of variant selection in surface martensite; however, the Jaswon and Wheeler distortion and the continuous distortion are more appropriate for accounting for other features of the transformation, such as the habit plane and accommodation by twin-related variant pairing.

## 2. Material and methods   

### 2.1. Experimental method   

An as-cast Fe–30% Ni alloy was cut and polished manually. The sample was prepared for electron backscatter diffraction (EBSD) mapping by electropolishing with the Struers A2 electrolyte (35 V, 20 s). Surface martensite appeared after electropolishing using an electrolyte at 283 K, meaning that the transformation took place approximately 10 K above the bulk martensitic start temperature. Maintained at the same temperature, the sample kept transforming hours later, which indicates an isothermal type of transformation. Optical images showing the evolution of the martensite transformation are available in §III of the supplementary material (Fig. S6). EBSD characterization was performed with an FEI-XLF 30 field emission gun scanning electron microscope using the Oxford Instruments acquisition software.

### 2.2. Predictive models for variant selection   

The EBSD measurements, presented in §3, show a significant variant selection even though the material was not subjected to any stress or strain, prior to or during transformation. The variant selection phenomenon is believed to result from the geometric anisotropy caused by the presence of the free surface, which allows the martensite to expand, free of constraints, in the direction normal to the surface. Predictive models based on this observation and considering different modelling of the transformation are proposed.

#### 2.2.1. Modelling of the transformation   

Various models are available in the literature (Bowles & Mackenzie, 1954 ▸; Jaswon & Wheeler, 1948 ▸; Cayron, 2015 ▸). Here, we will consider different descriptions to compare them and analyse which is the most appropriate to predict variant selection in surface martensite. The mathematical descriptions of the transformation are defined by a transformation matrix 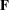. 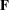 gives the image by the transformation of any vector 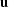: 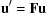. Four descriptions, expressed in the austenite crystallographic basis, are considered in this work:

*The Jaswon and Wheeler distortion.* The Kurdjumov–Sachs orientation relationship 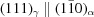; 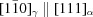 is often found in martensitic steels (Kurdjumow & Sachs, 1930 ▸). On the basis of this orientation relationship, a face centred cubic–body centred cubic (f.c.c.–b.c.c.) distortion has been proposed by Jaswon & Wheeler (1948 ▸). As a function of the lattice parameters, the distortion can be written as
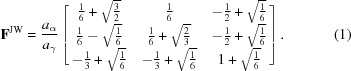
For Fe–30% Ni, we consider the lattice parameters 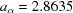
 Å and 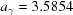
 Å for martensite and austenite, respectively, as measured by Goldman & Wagner (1963 ▸).

*The continuous distortion*. A continuous distortion for the f.c.c.–b.c.c. transformation has been recently proposed (Cayron, 2015 ▸). This continuous distortion, denoted 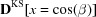 by the aforementioned author, leads to the Kurdjumov–Sachs orientation relationship. It can be expressed using a unique angular parameter *x* which evolves during the lattice transformation:
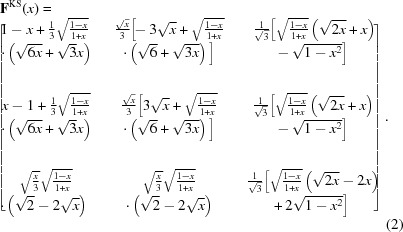
The two extremal *x* values are 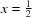 and 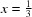, for the initial and the final distortion of the f.c.c. lattice, respectively. It is worth recording that the complete distortion 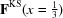 is equal to the Jaswon & Wheeler distortion with lattice parameters in a hard-sphere ratio, *i.e.* 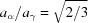.

*PTMC invariant plane strain.*. Variant selection caused by applied stress is classically studied using the invariant plane strain (IPS) computed from the phenomenological theory of martensite crystallography (Bhadeshia *et al.*, 2008 ▸). Here, the IPS is calculated with the *PTCLab* software (Gu *et al.*, 2016 ▸), using the Goldmann and Wagner lattice parameters for Fe–30% Ni and assuming twinning on 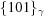 as lattice invariant shear (details are given in §V of the supplementary material). From the two solutions computed using the PTMC, the one that best fits the experimental data is chosen.
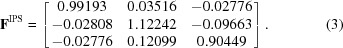



*PTMC invariant line strain.*. Beside the IPS, the PTMC also allows computation of the invariant line strain (ILS) associated with the phase transformation. For our alloy, the invariant line strain is given by
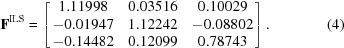



#### 2.2.2. Variant selection criteria   

A variant selection criterion is then associated with each description of the transformation. For the invariant line strain 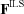, the invariant plane strain 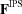 and the Jaswon and Wheeler 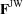 models, the criterion is the maximization of the extension of a material fibre oriented along the normal to the free surface. The term ‘material fibre’ comes from continuum mechanics and describes a set of material particles oriented along a given direction. It is not related to any texture consideration. This criterion can also be seen as the maximization of the volume change intrinsic to the transformation in a direction normal to the surface. To express it mathematically, we need to consider the gradient of displacement 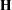 associated with the phase transformation 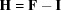. The displacement associated with the extension of a material fibre oriented along the normal to the free surface is given by the magnitude of 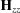 when 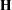 is expressed in the sample reference basis 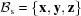. A sightly different criterion is chosen for the continuous model. Taking advantage of the continuous description, we propose to consider the maximization of the *variation* of the extension of a material fibre oriented along the normal to the free surface, at the beginning of the transformation. We thus need to compute the derivative 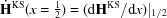 of the continuous distortion and evaluate the magnitude of 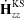. The criterion based on the maximization of 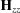 in the case of the continuous model has also been tested in this study, but since the predictions agree with those obtained using the Jaswon and Wheeler distortion they are not presented here. For the variant selection, a threshold based on the absolute value of the magnitude of 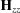 or 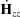 is set to best fit the experimental data in each of these four cases. The 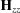 threshold values are 0 for the Jaswon and Wheeler model and the PTMC invariant line strain and 0.03 for the invariant plane strain. The 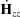 threshold is set to 0.2. The predictive models have been implemented using the *MTEX* toolbox (Bachmann *et al.*, 2010 ▸).

## 3. Results and discussion   

### 3.1. Experimental evidence of variant selection   

**Figure 1:**
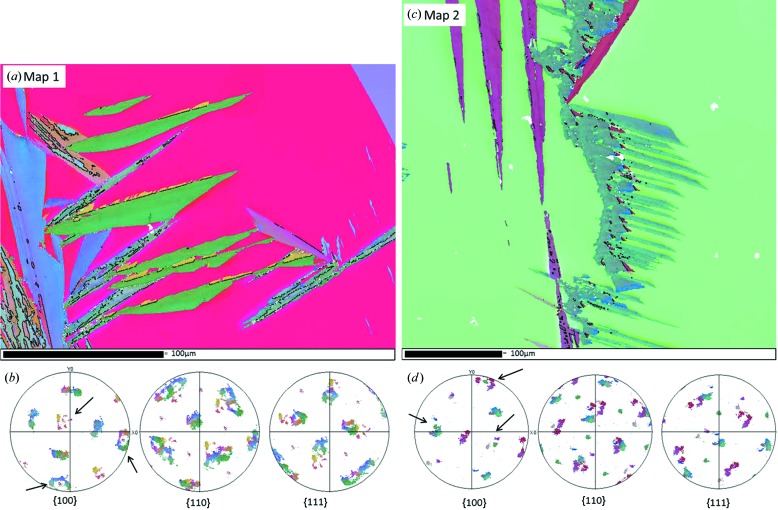
EBSD measurements of surface martensite, coloured by Euler coding. (*a*), (*c*) EBSD maps 1 and 2. (*b*), (*d*) Pole figures of the martensite in maps 1 and 2, respectively.

Fig. 1 ▸ shows two EBSD maps of surface martensite and the corresponding pole figures of the martensite. Owing to the large grain size in the sample, up to millimetre scale, these maps only show a part of the parent grain. Therefore, a lower-magnification EBSD measurement has been performed to verify that the measurement of a single ‘island’ of martensite is representative of the transformation produced in the whole grain. The lower-magnification map is presented in the supplementary material in §I. From the pole figures 1 ▸(*b*) and 1 ▸(*d*), a pronounced variant selection can be observed. In these two examples, the selection relates to variant grouping in so-called ‘Bain packets’ (Cayron, 2013 ▸). In the martensitic transformation, there exist three Bain packets, each of them containing eight variants which all have the same Bain strain. In 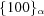 pole figures, Bain packets are easily recognizable by the circles around the 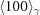 poles. They are indicated by arrows in Figs. 1 ▸(*b*) and 1 ▸(*d*). It seems that the variants belonging to Bain packets having a contraction axis oriented close to the normal to the surface, *i.e.* having an expansion located in the plane parallel to the surface, appear more weakly than the others. In map 1, two Bain circles are complete, whereas the third one is almost missing. In map 2, one Bain circle is almost complete and the other two only appear partially.

### 3.2. Variant selection predictions   

**Figure 2:**
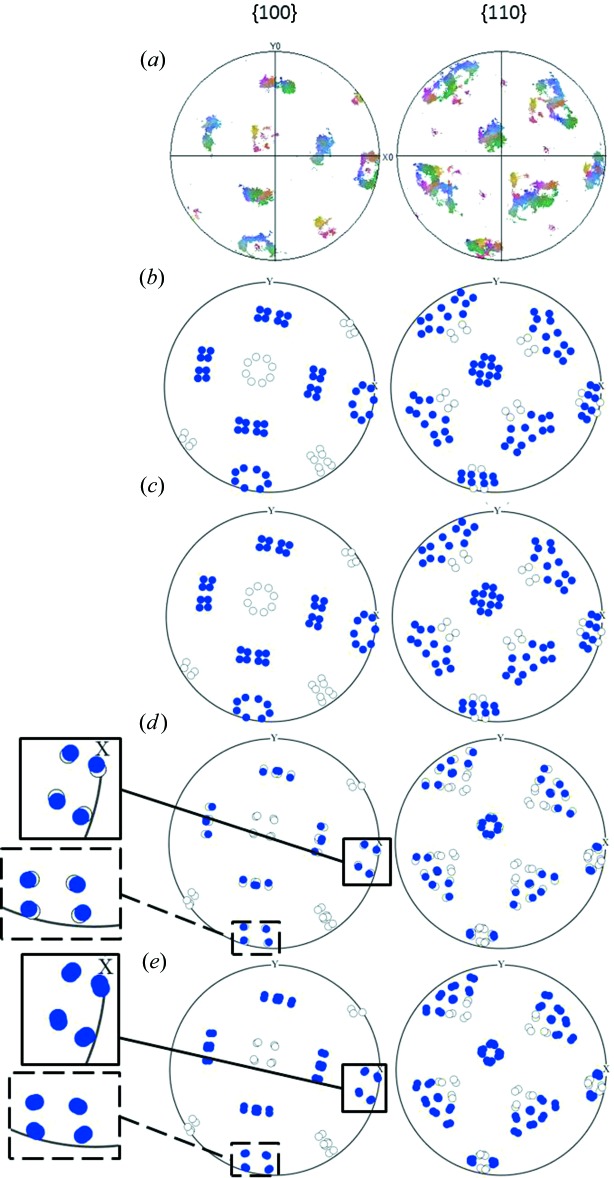
Prediction of variant selection for map 1. Blue circles, the selected variants. Empty circles, unselected variants. (*a*) Measured pole figure. (*b*) Jaswon and Wheeler distortion. (*c*) Derivative of the continuous distortion. (*d*) PTMC invariant plane strain. (*e*) PTMC invariant line strain.

Figs. 2 ▸ and 3 ▸ show the predictions of the variant selection according to the four criteria applied to maps 1 and 2, respectively. When needed, detailed views have been added on the pole figures in order to better visualize the predicted variants. From Figs. 2 ▸ and 3 ▸ it can be seen that the trend of the variant selection by Bain packets is predicted quite correctly with all four models. Additional EBSD measurements and associated simulations, available in §II of the supplementary material, show the same quality of predictions.

#### 3.2.1. Accommodation by twin-related variants   

The main discrepancy between the predictions – regardless of the model used – and the experiments is the light presence of the variants of the Bain packet that is associated with the compression along the surface normal. According to our computations, the variants belonging to this Bain packet are not favoured by the presence of a free surface, as for these variants the expansion mainly takes place in the *xy* plane. They are thus not selected in our predictive models. Nonetheless, we are able to explain their appearance by considering a ‘twinning’ accommodation mechanism of the habit plane. By their crystallographic nature, two twin-related variants belong to two different Bain packets (Cayron, 2013 ▸). Therefore, to accommodate the nucleation of all variants belonging to two specific Bain packets that are favoured by the free surface, one needs variants from the third Bain packet, even if this packet is not favoured. The EBSD map in Fig. 4 ▸(*a*) with the twin boundaries marked in black shows that the variants belonging to the unpredicted Bain circle (see Fig. 4 ▸ *b*) are twin related to the variant composing predominantly the martensite plates. It can be seen that the unfavoured twin-related variants are only located on the habit planes of the plates, which traces are indicated with dashed lines in Fig. 4 ▸(*a*), confirming their role in the accommodation mechanism.

#### 3.2.2. Derivative criterion *versus* complete distortion criterion   

**Figure 3:**
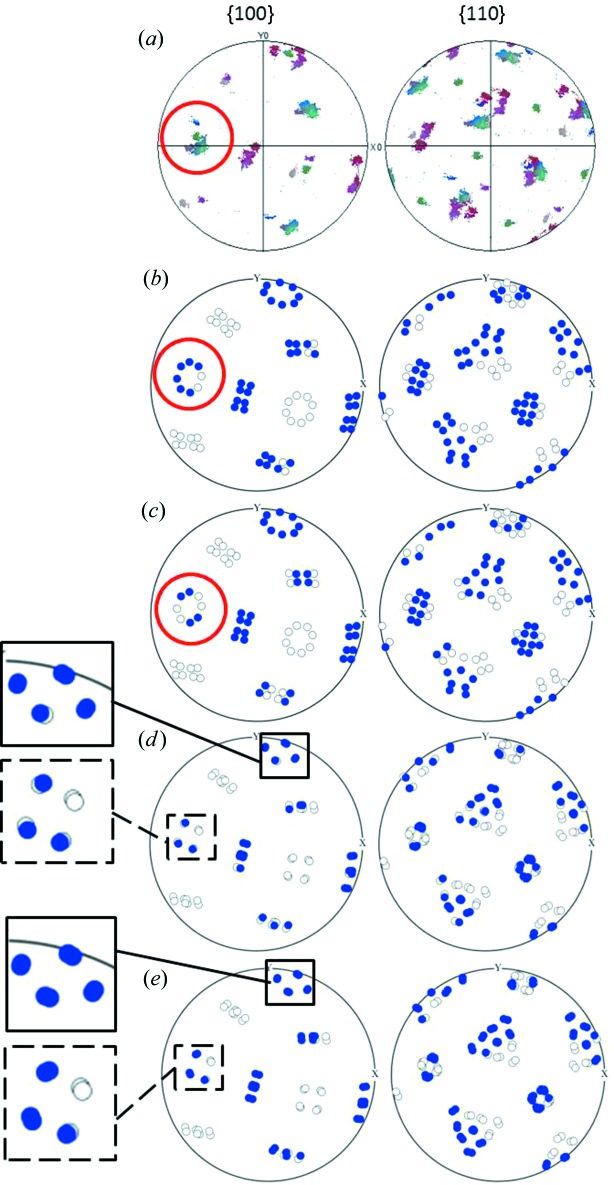
Prediction of variant selection for map 2. Blue circles, the selected variants. Empty circles, unselected variants. (*a*) Measured pole figure. (*b*) Jaswon and Wheeler distortion. (*c*) Derivative of the continuous distortion. (*d*) PTMC invariant plane strain. (*e*) PTMC invariant line strain.

As already mentioned, for the criterion based on the maximization of the extension of a material fibre along the normal to the free surface (max 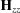), the continuous model and the Jaswon and Wheeler model give the same results. Thus, it is worth comparing Figs. 3 ▸(*c*) and 3 ▸(*b*) with Fig. 3 ▸(*a*) in order to analyse the effect of considering a criterion based on the variation of the extension (maximum 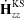), using the continuous distortion, instead of the criterion based on the total extension (maximum 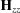), associated with the complete lattice distortion. By focusing on the Bain packet indicated with red circles, it seems that the criterion based on the variation improves the predictions for the variant distribution inside the indicated Bain packet, as compared to the complete distortion. This result might suggest that variant selection takes place at an early stage of the transformation, the variant being selected at the beginning of the lattice distortion. Such a view is quite in opposition to the idea of variant selection based on the invariant plane strain. Indeed, the IPS criterion includes the consideration of the full distortion along with the type of accommodation as relevant for the variant selection. In other words, in the derivative criterion, the early stage of the transformation is considered as crucial for the selection, whereas in the IPS criterion it is the final accommodated product which drives the selection.

#### 3.2.3. Quantitative comparison of the models   

To quantify and compare more properly the quality of the predictions, we performed a computational image comparison of the predicted and the experimental data. Binary images of the experimental and simulated pole figures are superimposed and the number of pixels that are correctly predicted, *i.e.* that are common in both the images, are counted. This number is then normalized by the union of the predicted and simulated pixels. The computational comparison is based on the 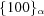 and 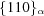 pole figures for both maps. The results are presented in Table 1 ▸. Details of the quantitative comparison of the variant selection are available in the supplementary material in §VI. A second method, consisting of computing the angular deviation of each experimentally measured martensite grain with respect to the closest predicted martensitic variant, has also been used. The results were not substantially different from those presented above. Both comparisons show that the trend of variant selection is captured by all models and that we cannot completely discriminate one model from another on the quality of variant selection prediction.

#### 3.2.4. Orientation relationship and habit plane predictions   

**Figure 4:**
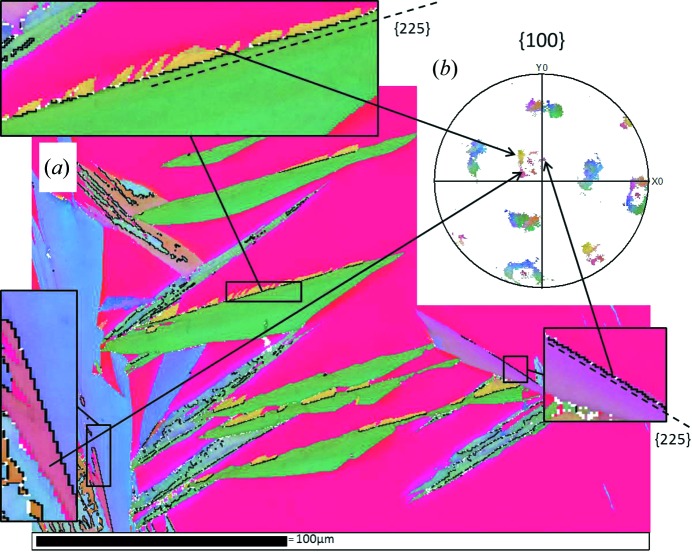
Twin-related variant combination for habit plane accommodation. (*a*) EBSD map, with twin boundaries marked in black and the 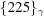 habit plane indicated with dashed lines. (*b*) 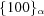 pole figure.

**Figure 5:**
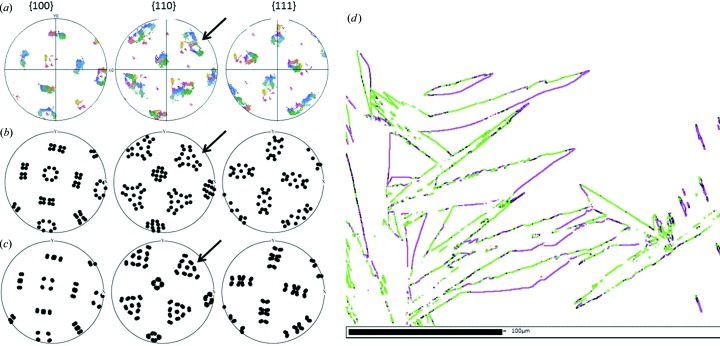
Orientation relationships. (*a*) Experimental pole figures. (*b*) Pole figures of the Jaswon and Wheeler and continuous distortions (KS). (*c*) Pole figures from the PTMC calculations. (*d*) Colour map of interphase boundaries, in green, within 3° of KS, and in magenta, within 3° of NW.

Besides the variant selection phenomenon, it is also worth comparing the quality of the prediction for other features of the transformation, such as the orientation relationship (OR) and the habit plane. The nature of the OR can be deduced from the shape of the continuous patterns in the martensite pole figures (Suikkanen *et al.*, 2011 ▸). In particular, the curvature of the outer contour of threefold stars in the 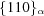 pole figure tells us whether the OR is closer to that of Nishiyama–Wassermann (NW) or to that of Kurdjumov–Sachs (KS). In Figs. 5 ▸(*a*)–5 ▸(*c*), arrows indicate the part of the pattern to compare. If the external contour of the star is convex as is the case in Fig. 5 ▸(*c*), the OR is close to that of Nishiyama–Wassermann, and if it is concave as in Fig. 5 ▸(*b*), it is close to that of Kurdjumov–Sachs. By inspection, the measured pole figures indicate that the OR is closer to that of Kurdjumov–Sachs. The measured OR is quite surprising as the NW OR was originally observed in bulk martensite formed in Fe–30% Ni and the same OR was estimated in surface martensite by Klostermann & Burgers (1964 ▸). A closer analysis of the OR based on the misorientations across f.c.c.–b.c.c. boundaries has been carried out. Fig. 5 ▸(*d*) shows the interphase boundaries associated with map 1. The boundaries are marked in green when they are close to the KS OR and in magenta when they are close to the NW OR. It can be observed that near the habit plane the OR is closer to KS, but as the martensite plate grows, the OR tends to get closer to the NW one. The same type of gradient in the orientation relationship has already been observed by Sato & Zaefferer (2009 ▸) in butterfly martensite. They measured an OR close to Kurdjumov–Sachs near the 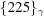 outer interface of the butterfly wings, while the Greninger–Troiano OR and the Nishiyama–Wassermann OR were found along the inner interface. To estimate quantitatively the occurrence of each OR in surface martensite, we measured the length of the boundaries associated with the two respective ORs. In agreement with the results of analysing the shape of the pole figure, boundaries close to KS are more often found than boundaries close to NW. Some additional details of this analysis, including the measurement of the bulk OR (Fig. S17), are proposed in §VIII of the supplementary material. The type of habit plane is another relevant feature of the transformation that needs to be accounted for in the transformation models. From the analysis of EBSD maps (supplementary material §IV), we found that the habit plane is of type 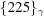. This result has already been reported in the literature (Wakasa & Wayman, 1979 ▸; Klostermann & Burgers, 1964 ▸). The crystallography of the habit planes thus disagrees with the PTMC calculations which predict a habit plane close to 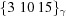. On the other hand, the Jaswon and Wheeler distortion and the continuous distortion can account for untilted 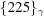 (Jaswon & Wheeler, 1948 ▸; Cayron, 2015 ▸). In the continuous model, the 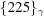 habit plane even becomes fully invariant by pairing the variants that are twin related (Baur *et al.*, 2017 ▸). In this configuration, the associated shape deformation is exactly an IPS on 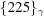. This type of accommodation agrees particularly well with Klostermann’s observations of isothermal martensite growth (Klostermann, 1972 ▸). According to Klostermann’s study, the initial stage of the plate growth is an IPS on 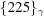. The plate then grows transversely and the associated shape deformation becomes inhomogeneous. To fully appreciate the agreement between Klostermann’s observations and the continuous distortion, it is worth considering again Fig. 4 ▸. This shows that an energetically unfavoured variant twin related to a favoured one is needed to accommodate the transformation on the habit plane. The combination of these variants on the habit plane produces an IPS. This corresponds to Klostermann’s first stage. Then, once the habit plane is accommodated, the twin that is energetically most favoured grows preferentially. Both the Jaswon and Wheeler distortion and the continuous model are able to account convincingly for the accommodation mechanism and the habit plane observed in surface martensite. Using the PTMC, the surface martensite habit plane is wrongly predicted if the real lattice parameters are considered, but one can account for such a habit plane by using lattice parameters in a hard-sphere packing ratio as input for the computation (Baur *et al.*, 2017 ▸). In terms of orientation relationship, it appears that the Jaswon and Wheeler distortion and the continuous distortion describe the early stage of the transformation well, while PTMC prediction of the orientation relationship is valid for the fully grown plate.

## 4. Conclusion   

In conclusion, we observed and analysed variant selection in surface martensite formed in an Fe–30% Ni alloy. The variant selection was found to be related to the Bain packets. A prediction model based on the maximization of the extension of a material fibre oriented along the normal to the free surface is proposed. Different descriptions of the transformation have been tested. In particular, PTMC matrices, namely the invariant plane strain and the invariant line strain, the Jaswon and Wheeler distortion, and the continuous distortion, have been compared. The variant selection prediction is not significantly different using these different models, such that none of the models can be discriminated from the others on this basis. However, the Jaswon and Wheeler distortion and continuous distortion allow a more complete description of the transformation as they account well for other features of the transformation such as the 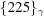 habit plane and the accommodation mechanism by twin-related variant pairing. Finally, this study suggests that it might be worth considering the *variation* of the extension of a material fibre oriented along the normal to the free surface at an early stage of the transformation, using the continuous description of the transformation, instead of its total extension based on the complete transformation.

## 5. Related literature   

For additional literature relating to the supporting information, see Nishiyama (1934 ▸), Wassermann (1933 ▸) and Cayron (2014 ▸).

## Supplementary Material

Supporting information file. DOI: 10.1107/S160057671701398X/nb5203sup1.pdf

## Figures and Tables

**Table 1:** Comparison of the quality (%) of the predictions for the four different models

	PTMC ILS	PTMC IPS	JW distortion	Continuous distortion
Map 1	19	14	21	21
Map 2	14	13	14	16
