# Supplementary material for: Variant selection in surface martensite
Source: J Appl Crystallogr. 2017 Oct 20;50(Pt 6):1646–52. doi: 10.1107/S160057671701398X (PMC5713143; doi:10.1107/S160057671701398X)
Supplement: Supplementary file 1 [file j-50-01646-sup1.pdf]

Variant selection in surface martensite: Supplementary material

Annick Baur, Cyril Cayron & Roland Loge

Laboratory of Thermomechanical Metallurgy, PX Group Chair
Ecole Polytechnique Fédérale de Lausanne
annick.baur@epfl.ch

I. Characterization at lower magnification

The EBSD measurements presented in the main text are showing "islands" of martensite formed in only a part of the parent grain. In order to verify that the variant selection occurring in a single island of martensite is representative of the transformation behaviour of the entire grain, a lower magnification EBSD characterization is proposed here.

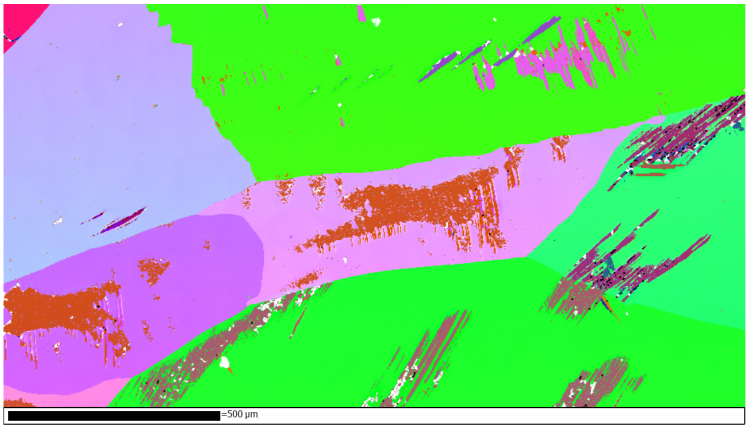

(a) Euler colour coding

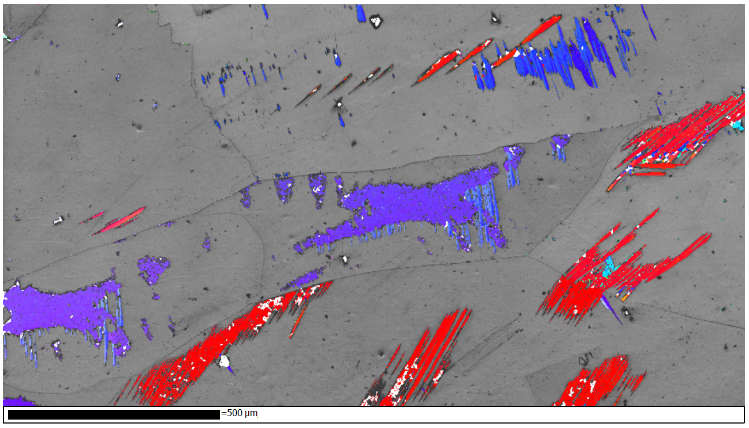

(b) Band contrast and Euler colour coding

Figure S 1: *EBSD map of surface martensite at lower magnification (x65).*

The grain size in the sample is large, and grains can reach the millimetric size. From this

measurement, it can be seen that all the islands of martensite formed in a given grain are very similar, which allows us to consider only one of them as representative of the whole. The analysis of the variant selection phenomenon proposed in the main manuscript is thus based on the measure at higher magnification (x500) of few of these islands.

II. Additional simulations

EBSD measurements

Additional simulations have been performed based on different EBSD maps acquired from the same as-cast Fe30%Ni sample. Figures S2 and S3 show the two additional maps and their respective pole figures of the BCC phase.

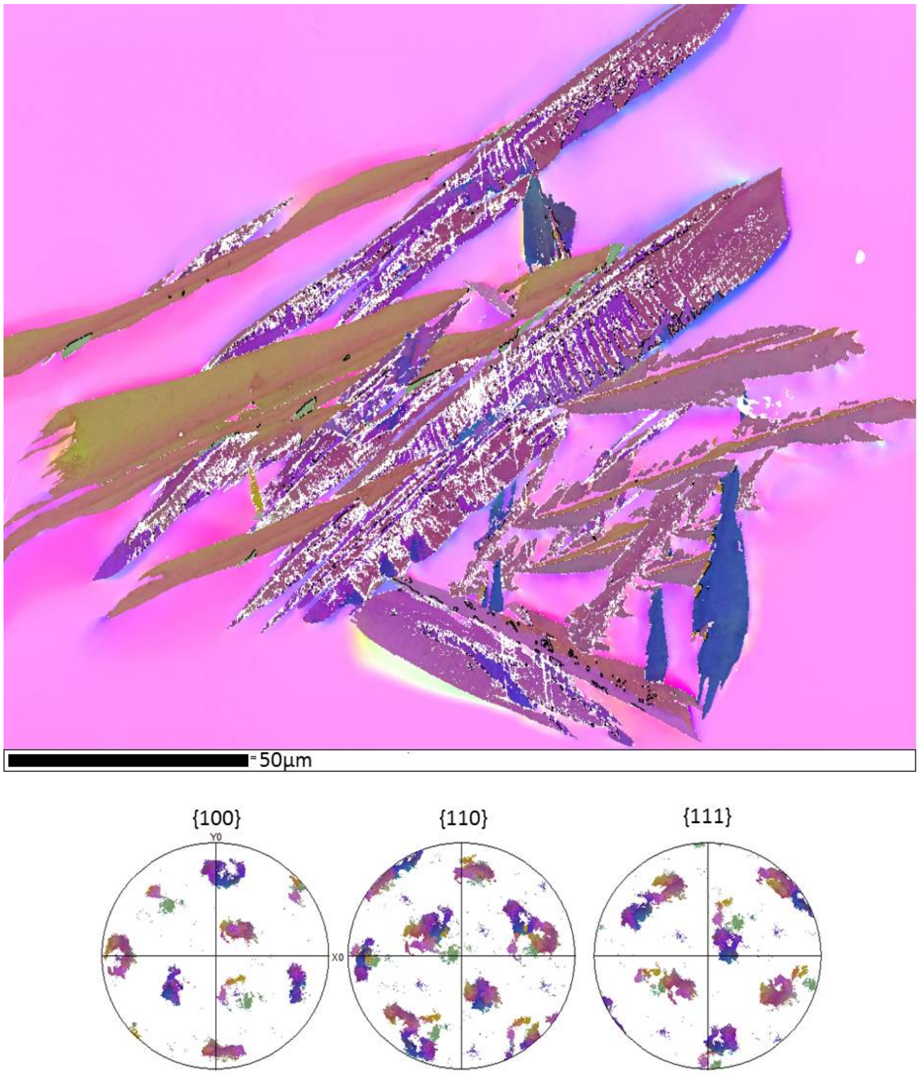

Figure S 2: *EBSD measurements of surface martensite and pole figures of the martensite.*

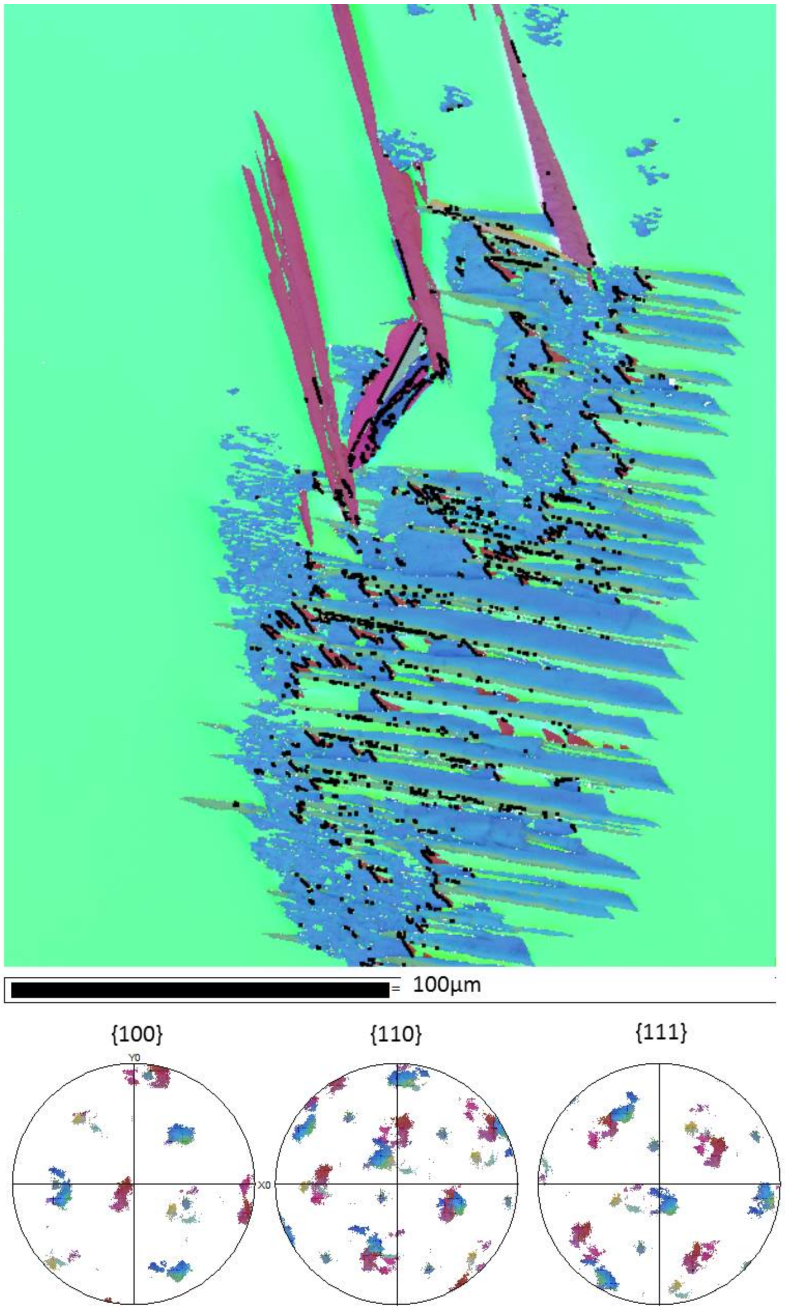

Figure S 3: *EBSD measurements of surface martensite and pole figures of the martensite.*

Variant selection predictions

Using the same criterion as in the article, the prediction of the variant selection is presented in figures S4 and S 5 for the first and the second additional maps, respectively.

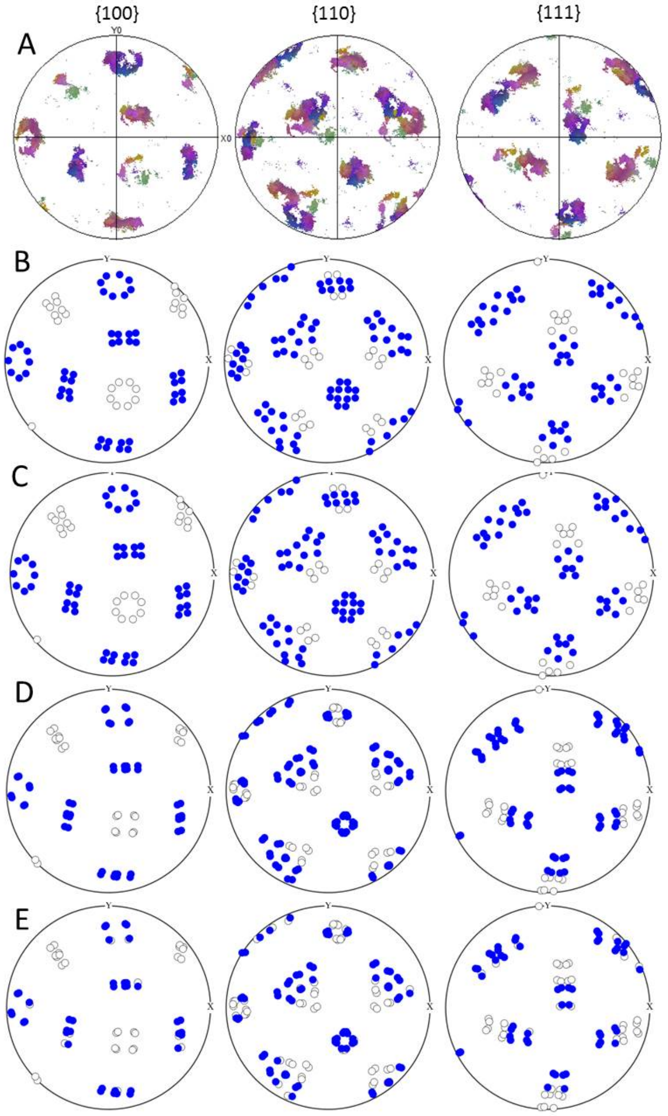

Figure S 4: *Prediction of variant selection for the map 1. In blue, the selected variants. Empty circles, unselected variants.* ***A****: Measured pole figure.* ***B****: Kurdjumov-Sachs distortion.* ***C****: Derivative of the Kurdjumov-Sachs distortion.* ***D****: PTMC invariant plane strain.* ***E****: PTMC invariant line strain.*

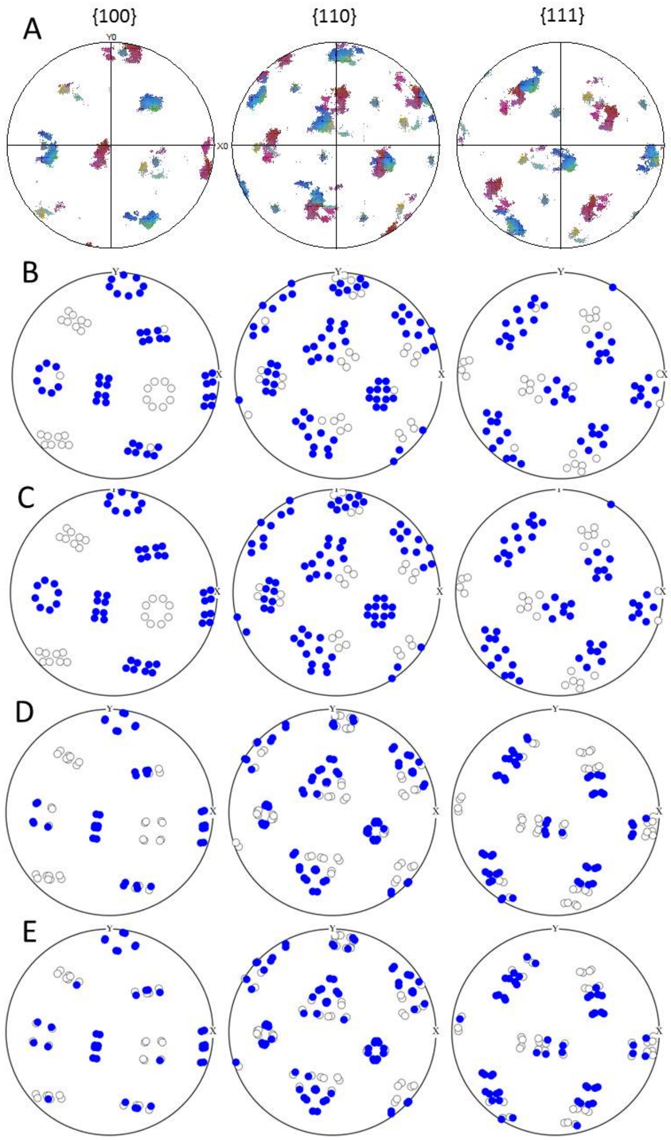

Figure S 5: *Prediction of variant selection for the map 2. In blue, the selected variants. Empty circles, unselected variants.* ***A****: Measured pole figure.* ***B****: Kurdjumov-Sachs distortion.* ***C****: Derivative of the Kurdjumov-Sachs distortion.* ***D****: PTMC invariant plane strain.* ***E****: PTMC invariant line strain.*

III. Isothermal surface martensite

Directly after electro-polishing and some days later, micrograph pictures have been taken showing the presence of surface martensite. These respective pictures are presented in figure S 6a and 6b.

By comparing the two micrographs, it can be observe that new martensite plates continue to appear even though the temperature is constant (the sample stayed in the fridge at the same temperature as the electrolyte). The surface martensite observed is thus *isothermal* martensite.

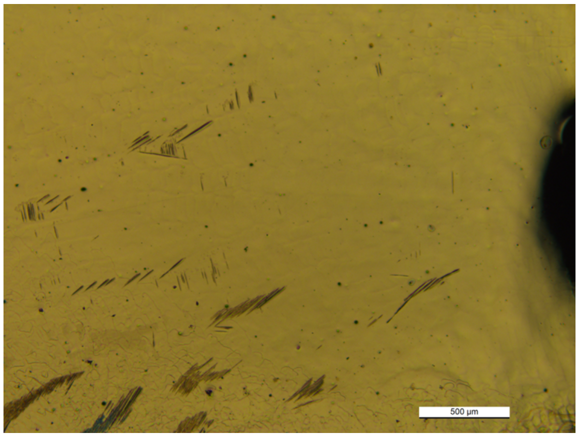

(a) After polishing

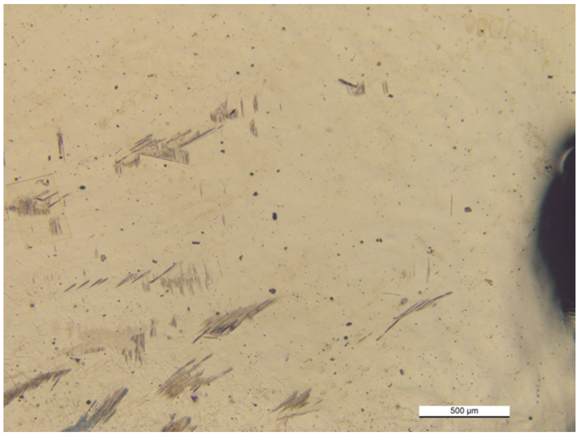

(b) A few days after polishing

Figure S 6: *Bright field micrographs of surface martensite*

IV. Crystallography of the habit plane

Comparison between typical habit planes

Using PTMC, the habit plane of Fe30%Ni is predicted to be close to $\{3\,10\,15\}_\gamma$ and this prediction is generally experimentally verified, *in the bulk*. However, in the surface martensite observed by Klostermann (1972) and later by Wakasa and Wayman (1979) the habit plane is of type $\{225\}_\gamma$. In our sample, we studied the crystallography of habit plane by verifying whether the traces of the habit planes visible on the EBSD maps correspond to $\{3\,10\,15\}_\gamma$ or to $\{225\}_\gamma$. Figures S7a and S7b present the results of this study for the $\{3\,10\,15\}_\gamma$ and $\{225\}_\gamma$ habit planes, respectively.

By comparing figures S7a and S7b, it appears that the habit plane is not of type $\{3\,10\,15\}_\gamma$ but is likely to be of type $\{225\}_\gamma$. Indeed, figure S 7a shows that for several plates of martensite, the normal to the trace of the habit plane does not intersect any $\{3\,10\,15\}_\gamma$ poles, except the pole oriented along the z-direction. However, these plates have different habit planes one to another, and by inspection none has a habit plane normal to z. This means that the habit plane cannot be of type $\{3\,10\,15\}_\gamma$. Figure S 7b, on the contrary, shows the study of the $\{225\}_\gamma$ habit plane case.

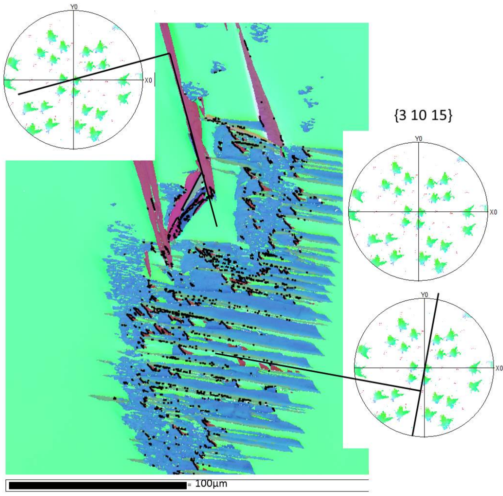

(a) $\{3\ 10\ 15\}_\gamma$ case

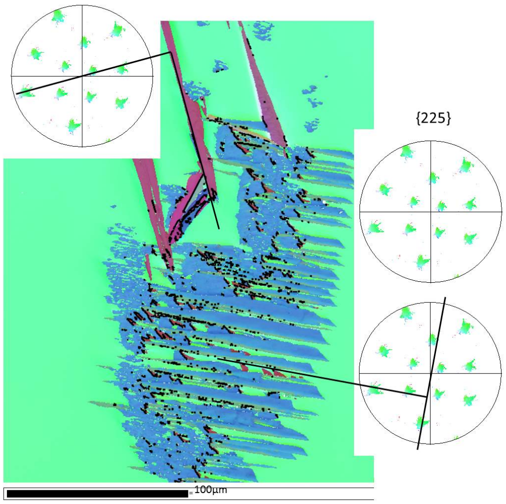

(b) $\{225\}_\gamma$ case

Figure S 7: *Study of the habit plane of surface martensite*

Here, it can be observed that each plate can be associated to a $\{225\}_\gamma$ pole. Surface martensite in Fe30%Ni seems therefore to have $\{225\}_\gamma$ habit planes.

Verification of the choice of the habit plane trace

Figure S 8 shows the misorientation in the austenite around the martensite plates in respect with the austenite orientation far from the plates.The red colour means that the misorientation is about 7° and the blue colour indicate that there is no misorientation. This allows to get convinced that the traces of the habit plane have been chosen in an appropriate way, as the distortion of the austenite near this region is weaker than anywhere else on the border of the plate.

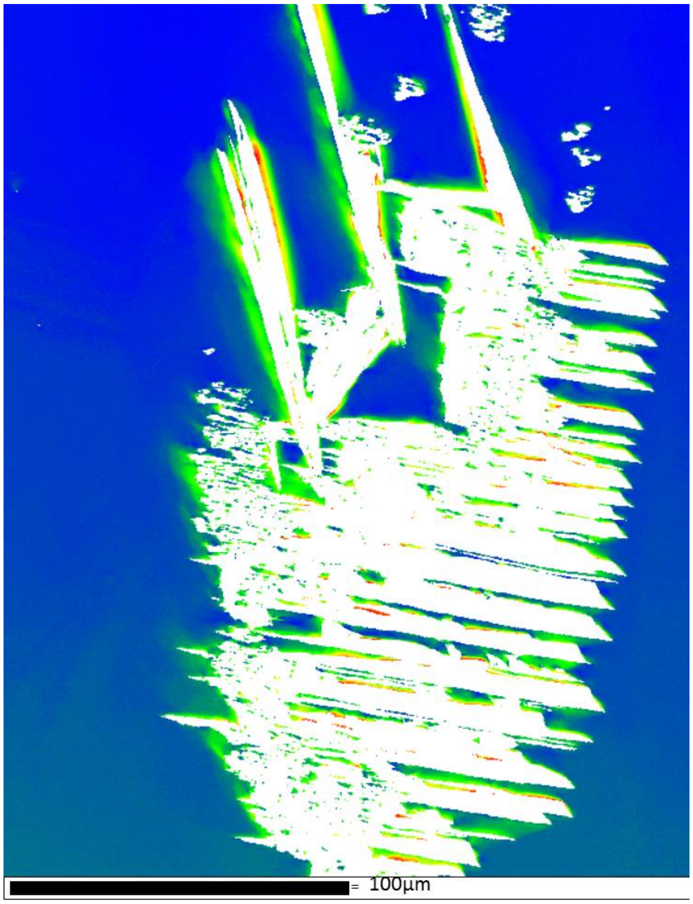

Figure S 8: *EBSD measurements of surface martensite: Internal misorientation of the austenite. Blue colour, no misorientation. Red colour, misorientation of 7°.*

V. The PTMC solutions

The PTMC solutions for the Fe30%Ni have been computed thanks to the open source software *PTCLab*. The inputs for the computations are the lattice parameters of the FCC and the BCC phases, the correspondence matrix and the lattice invariant shear system. In our study, the Bain correspondance has been chosen and the lattice invariant shear is assumed to occur by twinning on $\{101\}_\gamma$. No dilatation parameter is considered, such that we pose $\delta = 1$. The lattice parameter of the alloy in the two different phases are respectivey: 3.5854Å for the austenite and 2.8635Å for the martensite. These lattice parameters are taken from Goldmann and Wagner (1963).
Figure S 9 shows the output of the *PTCLab* software, where four solutions are proposed.

Phase	a	b	c	alpha	beta	gamma	SG			Lattice C.	1.0	-1.0	0.0	Delta	1.0
Phase 1	3.5854	3.5854	3.5854	90	90	90	Fm-3m				1.0	1.0	0.0		
Phase 2	2.8635	2.8635	2.8635	90	90	90	Im-3m				0.0	0.0	1.0		
Condition:	SP:	1.0	0.0	1.0		SD:	-1.0	0.0	1.0						
Solution 1				Solution 2				Solution 3				Solution 4			
IL(in 1)	-0.65745	IL(in 2)	-0.23105	IL(in 1)	-0.65745	IL(in 2)	-0.81909	IL(in 1)	-0.65745	IL(in 2)	-0.23105	IL(in 1)	-0.65745	IL(in 2)	-0.81909
	-0.36814		-0.81909		0.36814		-0.23105		-0.36814		-0.81909		0.36814		-0.23105
	0.65745		0.52507		0.65745		0.52507		0.65745		0.52507		0.65745		0.52507
IL*(in 1)	0.52507	IL*(in 2)	0.74804	IL*(in 1)	0.52507	IL*(in 2)	0.74804	IL*(in 1)	0.52507	IL*(in 2)	-0.09059	IL*(in 1)	0.52507	IL*(in 2)	-0.09059
	-0.66977		-0.09059		-0.66977		-0.09059		0.66977		0.74804		0.66977		0.74804
	0.52507		0.65745		0.52507		0.65745		0.52507		0.65745		0.52507		0.65745
RB	1.09818	0.12099	0.16593	RB	1.11998	0.03516	0.10029	RB	1.11998	-0.03516	0.10029	RB	1.09818	-0.12099	0.16593
	-0.11087	1.12242	-0.04232		-0.01947	1.12242	-0.08802		0.01947	1.12242	0.08802		0.11087	1.12242	0.04232
	-0.23961	0.03516	0.78008		-0.14482	0.12099	0.78743		-0.14482	-0.12099	0.78743		-0.23961	-0.03516	0.78008
OR Mat.	0.61177	-0.77211	-0.17202	OR Mat.	0.67915	-0.71489	-0.16641	OR Mat.	0.72318	-0.6905	-0.01492	OR Mat.	0.76327	-0.63328	-0.12799
	0.76327	0.63328	-0.12799		0.72318	0.6905	-0.01492		0.67915	0.71489	-0.16641		0.61177	0.77211	-0.17202
	0.20776	-0.05299	0.97674		0.12557	-0.11022	0.98594		0.12557	0.11022	0.98594		0.20776	0.05299	0.97674
HP(in 1)	-0.60978	HP(in 2)	-0.93902	HP(in 1)	-0.17721	HP(in 2)	-0.57113	HP(in 1)	-0.17721	HP(in 2)	0.41436	HP(in 1)	-0.60978	HP(in 2)	0.04647
	0.77251		0.04647		0.77251		0.41436		-0.77251		-0.57113		-0.77251		-0.93902
	-0.17721		-0.34072		-0.60978		-0.7086		-0.60978		-0.7086		-0.17721		-0.34072
Macro s	0.68873	m2	0.22741	Macro s	-0.20015	m2	-0.22741	Macro s	-0.20015	m2	-0.22741	Macro s	0.68873	m2	0.22741
	0.69684				-0.69684				0.69684				-0.69684		
	0.20015				-0.68873				-0.68873				0.20015		
F	0.90449	0.12099	-0.02776	F	0.99193	0.03516	-0.02776	F	0.99193	-0.03516	-0.02776	F	0.90449	-0.12099	-0.02776
	-0.09663	1.12242	-0.02808		-0.02808	1.12242	-0.09663		0.02808	1.12242	0.09663		0.09663	1.12242	0.02808
	-0.02776	0.03516	0.99193		-0.02776	0.12099	0.90449		-0.02776	-0.12099	0.90449		-0.02776	-0.03516	0.99193
m1	0.41552			m1	0.25114			m1	0.25114			m1	0.41552		
OR	in 1	in 2	Angle	OR	in 1	in 2	Angle	OR	in 1	in 2	Angle	OR	in 1	in 2	Angle
Plane	1 -1 1	1. 0. 1.	0.60093	Plane	1 -1 1	1. 0. 1.	0.60093	Plane	1 1 1	0. 1. 1.	0.60093	Plane	1 1 1	0. 1. 1.	0.60093
Direction	1 0 -1	1. 1. -1.	3.82625	Direction	1 1 0	0. 2. 0.	1.57542	Direction	1 -1 0	2. 0. 0.	1.57542	Direction	1 0 -1	1. 1. -1.	3.82625

Figure S 9: *Chart of the PTMC solutions computed from* PTCLab

We can see that there exist only two crystallographically different solutions, as solutions 1 is equivalent to solution 4, and solution 2 equivalent to solution 3. Figure S 10 shows the pole figures of the martensite associated to the two different solutions.

It can be seen that the shapes of the patterns in the pole figures are relatively different in the two solutions. In the main article, solution 2 is chosen as it better fits the experimental data in terms of orientation relationship and variant selection prediction. In order to offer a comprehensive study, in this section, we present the results of the simulations using solution 1.

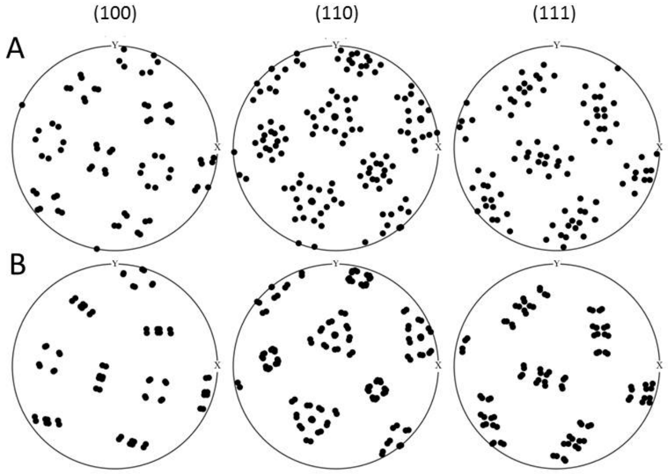

Figure S 10: *Martensite pole figure of the two PTMC solutions. **A**: Solution 1. **B** Solution 2.*

Simulations for the other solution

For the simulation, the same criterion as the ones imposed in the main article have been chosen. The results for map 1 and map 2 of the article are illustrated in figures S 11 and 12, respectively.

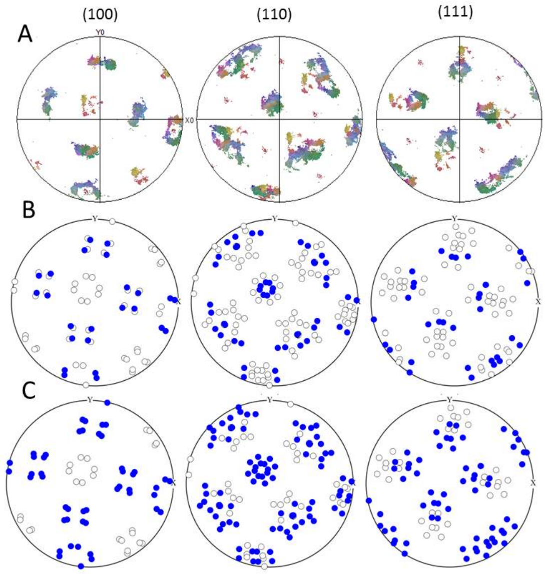

Figure S 11: *Prediction of variant selection for the map 1. In blue, the selected variants. Empty circles, unselected variants. **A**: Measured pole figure. **B**: PTMC invariant plane strain. **C**: PTMC invariant line strain.*

By inspection, it can be noted that according to this solution, the invariant strain line allows a slightly better prediction for the variant selection than the invariant plane strain.

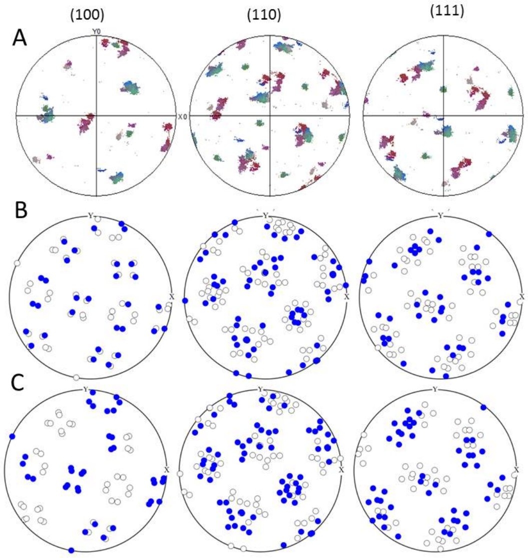

Figure S 12: *Prediction of variant selection for the map 2. In blue, the selected variants. Empty circles, unselected variants. **A**: Measured pole figure. **B**: PTMC invariant plane strain. **C**: PTMC invariant line strain.*

VI. Quantitative analysis of the variant selection prediction

The quality of the predictive models for variant selection is generally performed qualitatively, by comparing visually the experimental and the simulated pole figures. We propose a method based on image processing to quantify more properly the quality of the predictions. This method is briefly explained in this supplementary material.

Method

The quantitative measure of the quality of the prediction is based on pole figures analysis. The concept is to superimpose binary image of the predicted and the experimental pole figure. The measure of the quality is then performed by counting the number of pixels that are correctly predicted. In other words, we count the number of black pixels that are present in both the experimental and the predicted pole figures. This value is then normalized by the union of the predicted pixels and the experimentally measured pixels.

Comparison of the prediction for the various models

Figures S13 and S14 show the superimposition of the experimental and measured $\{100\}_\alpha$ and $\{110\}_\alpha$ pole figure for the PTMC invariant line strain, the PTMC invariant plane strain, the Jaswon & Wheeler distortion and the continuous distortion for map 2.

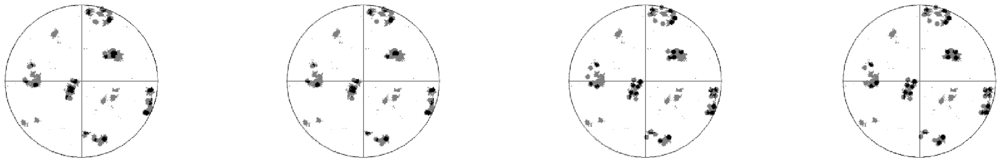

(a) PTMC ILS (b) PTMC IPS (c) JW distortion (d) Continuous distortion

Figure S 13: *Superimposition of the experimental and predicted* $\{100\}_\alpha$ *pole figures of map 2: In black, common pixels; in grey, pixels written in one of the two pole figures.*

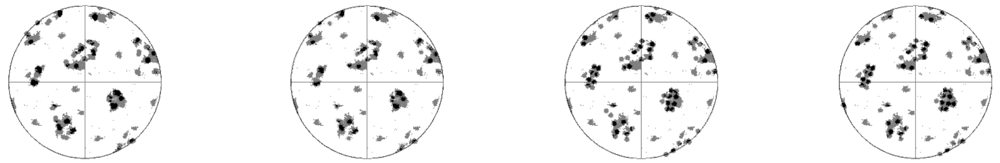

(a) PTMC ILS (b) PTMC IPS (c) JW distortion (d) Continuous distortion

Figure S 14: *Superimposition of the experimental and predicted* $\{110\}_\alpha$ *pole figures of map 2: In black, common pixels; in grey, pixels written in one of the two pole figures.*

VII. The PTMC solution with hard sphere ratio

We recently show (Baur *et al.*, 2017) that if one imposes the lattice parameters in a hard-sphere ratio, i.e. $\frac{a_\gamma}{a_\alpha} = \sqrt{3/2}$, the continuous distortion associated with the Kurdjumov-Sachs OR and the PTMC calculations lead to the same results. More precisely, the continuous distortion is equal to the invariant line strain of the PTMC and the equibalanced combination of twin-related variant of the continuous distortion is equal to the invariant plane strain.
Our study shows that in the case of surface martensite, the PTMC with the *real* lattice parameters of the Fe-30%Ni does not predict neither the correct orientation relationship nor the correct habit plane. Indeed, the habit plane measured is of type $\{225\}_\gamma$ and the OR is Kurdjumov-Sachs, whereas the PTMC predicts habit plane close to $\{259\}_\gamma$ and a Nishiyama OR. Imposing a hard sphere ratio as input to the PTMC, the calculations offers a correct prediction of these features. If we do so, the invariant line strain leads to a good variant selection prediction, exactly as the continuous distortion does. One could however wonder what would be the prediction of variant selection if one consider the shape deformation (IPS) instead. The results of such a prediction are presented in this supplementary.

Simulations based on the invariant plane strain

The shape deformation associated with a hard sphere lattice parameters ratio takes the following form in the crystallographic γ-basis.

$$\mathbf{F}^{IPS} = \begin{bmatrix} 0.9694 & -0.0306 & -0.0749 \\ -0.0306 & 0.9694 & -0.0749 \\ 0.0612 & 0.0612 & 1.1498 \end{bmatrix} \quad (1)$$

The calculation for the variant selection is conducted exactly in the same way as what is done in the main paper. The simulated pole figures as well as the experimental EBSD measurements are shown in figure S 15.

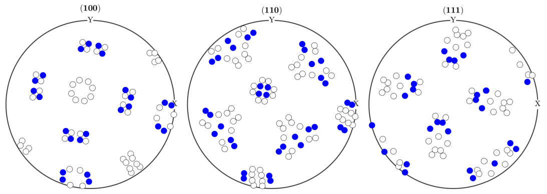

(a) Simulated map 1

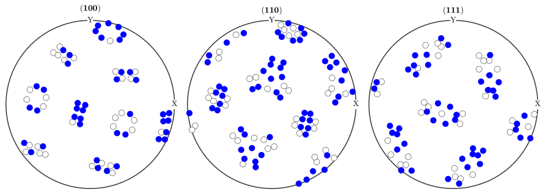

(b) Simulated map 2

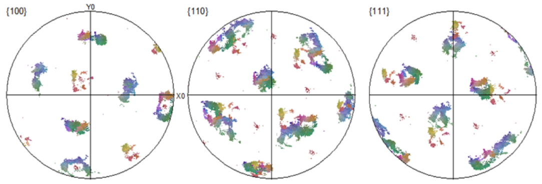

(c) Experimental map 1

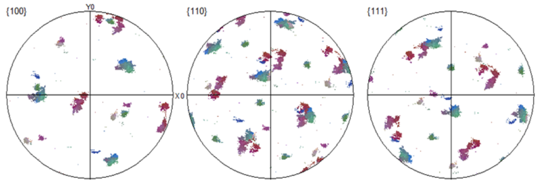

(d) Experimental map 2

Figure S 15: *Simulations based on the IPS associated with hard-sphere ratio*

Qualitatively, it can be noted that the prediction are not really convincing and that the prediction based on the invariant line strain (ILS) are better. For map 1 only, we analysed the quality of the predictions as described in the supplementary 5 and compare the result with the one obtained using the ILS. The results are presented in table 1. The associated superimposed $\{110\}_\gamma$ pole figures are shown in figure S 16.

Table 1: *Quality of the predictions with hard sphere lattice parameters*

	PTMC IPS	PTMC ILS
Quality	12%	21%

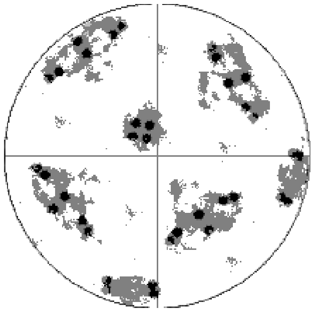

(a) Invariant Plane Strain

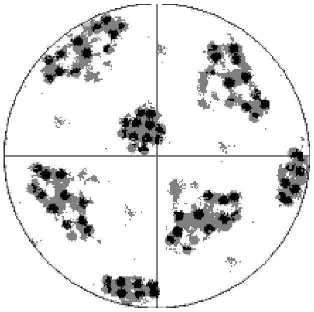

(b) Invariant Line Strain

Figure S 16: *Superimposition of the predicted and the measured* $\{110\}_\alpha$ *pole figure: In black, pixels that are written in the predicted pole figure* and *in the experimental measurement; in grey, pixels that are either predicted* or *measured*

VIII. Orientation relationships

The analysis of the orientation relationship in the surface martensite found in our Fe-30%Ni, based on the pole figures shape, indicates that it is closer to the Kurdjumov-Sachs OR than to the Nishiyama-Wassermann OR. As mentioned in the main text, this is quite surprising as it is with such an alloy that Nishiyama and Wassermann derived their famous OR (Nishiyama, 1934) (Wassermann, 1933), but both worked on bulk martensite and not on surface martensite. It seems therefore that the OR changes from a KS type to a NW type depending on whether the martensite is formed on the surface or in the bulk.
The same alloy as the one that presented surface martensite as been quenched in liquid nitrogen to form bulk martensite and the sample has then be prepared for EBSD mapping. The bulk martensite pole figure of a single grain of austenite is presented in figure S 17. It can be compared to figure S 18, the pole figure associated to the map 1 in the main manuscript.

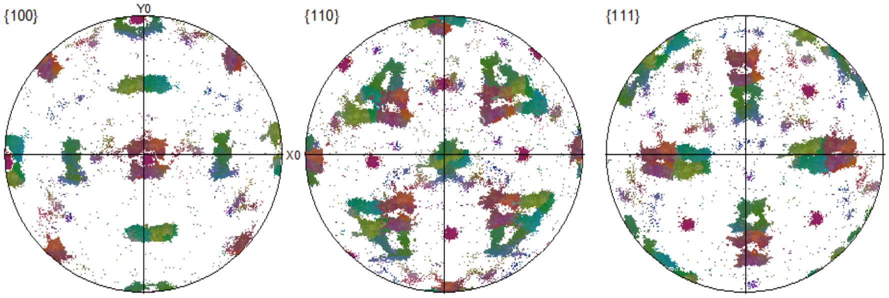

Figure S 17: *Pole figures of the bulk martensite*

The incurvation of the outer three-fold star in the {110} pole figure reveals that surface martensite is closer to KS than bulk martensite, as in the former the shape is tendencially more concave than in the latter where it is more convexe.

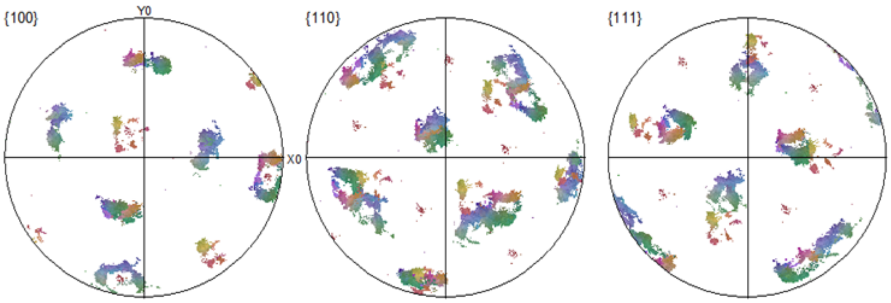

Figure S 18: *Pole figures of the surface martensite*

In order to assess more quantitatively the difference of OR, we used the HKL EBSD software to compute the misorientation on the interphase boundaries. On figure S 19 the interphase boundaries which show an misorientation within less than 3 degrees from the Kurdjumov-Sachs OR and the Nishiyama-Wasserman OR are indicated in green and magenta respectively. In black, are marked the boundaries that are at three degrees from both ORs.

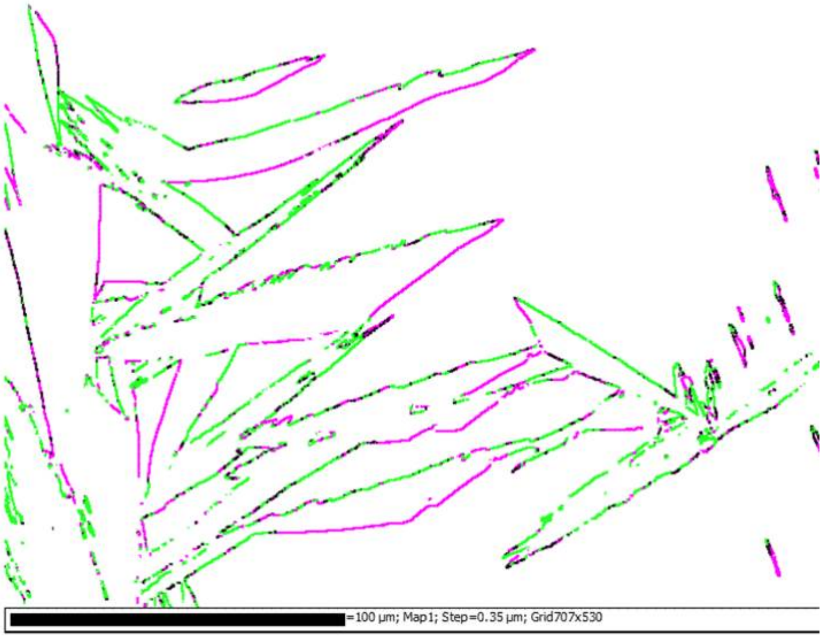

Figure S 19: *Orientation relationship at the grain boundaries. Green indicates the boundaries whithin* 3° *from KS and magenta indicates the one whithin* 3° *from NW.*

Figure S 19 seems to indicate that close to the habit plane the orientation relationship is of type Kurdjumov-Sachs, and that it moves towards a Nishyjama-Wasserman type of OR when the martensite plate grows. By counting the pixels associated to each type of boundary in both map 1 and 2, it is possible to assess to which OR the martensite formed is closer to. The number of pixels belonging in a interphase boundary that is associated to the KS OR (within 3°) and the

Table 2: *Comparison of the quality for the predictions for the four different models.*

	KS OR	NW OR
Map 1	64%	44%
Map 2	65%	47%

number of pixels belonging in a interphase boundary that is associated to the NW OR (within 3°) are counted and normalized by the total number of pixels defining the interphase boundary. The results are presented in table 2.

It can be noted that the sum of the percentage is greater than 100%, this is due to the fact that some boundaries are in the same time within 3° of NW and KS.

Figure S 20 shows the map of orientation relationship of the martensite plate based on the reconstruction of the parent austenite, using the method presented in reference [1]. This second

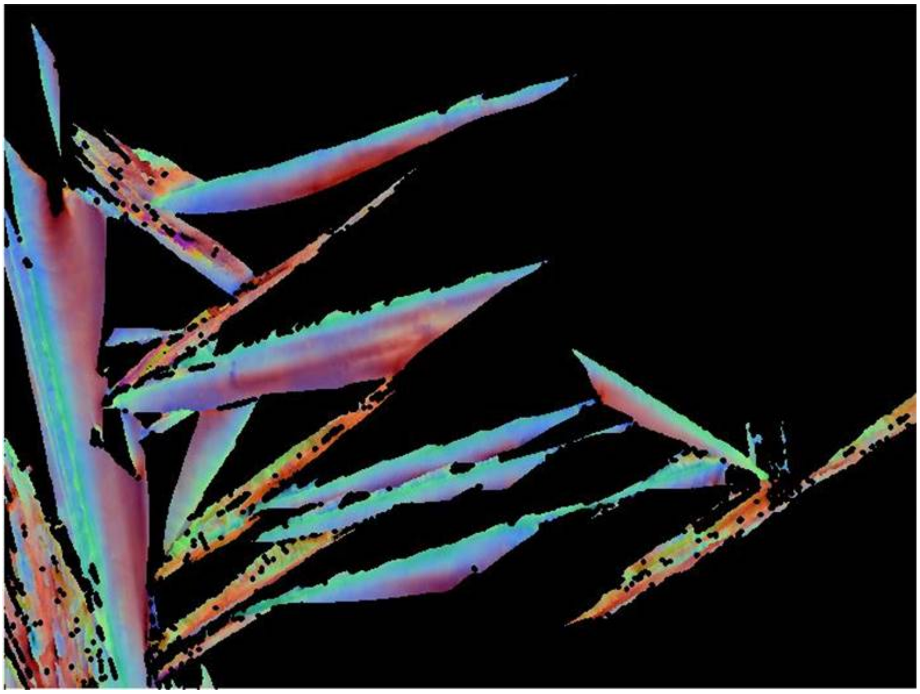

Figure S 20: *Orientation relationship in the plate. Color coding: KS in green, NW in blue and Pitsch in red.*

analysis confirms that the plate start to transform with a KS OR and far from the habit plane the OR changes towards NW OR and even locally towards Pitsch OR.

References

[1] Cayron, C. *EBSD imaging of orientation relationships and variant groupings in different martensitic alloys and Widmanstätten iron meteorites*, Material Characterization, **94**, 93-110, (2014).
